# Neurotoxicity, Cytotoxicity, and Genotoxicity of Phyto-radio Synthesized Selenium Nanoparticles in *Culex pipiens* Complex

**DOI:** 10.1007/s12011-024-04418-8

**Published:** 2024-10-24

**Authors:** H. H. Awad, I. Abulyazid, E. M. S. El-Kholy, H. S. Mohammed, H. K. Abdelhakim, A. M. Fadl

**Affiliations:** 1 Entomology Department, Faculty of Science, Cairo University, Giza, Egypt; 2 Biological Application Department, Nuclear Research Center, Egyptian Atomic Energy Authority, Cairo, Egypt; 3 Biophysics Department, Faculty of Science, Cairo University, Giza, Egypt; 4 Biochemistry Department, Faculty of Science, Cairo University, Giza, Egypt

**Keywords:** *Culex pipiens*, Detoxifying enzymes, Gene expression, Selenium nanoparticles, Gamma or microwave irradiation, Ultrastructure

## Abstract

Effective mosquito management strategies are crucial to minimize the number of mosquito-borne diseases. Selenium nanoparticles (SeNPs) are promising in mosquito control because they are effective and eco-friendly rather than synthetic insecticides. The current study was conducted to evaluate the impact of SeNPs on the detoxification enzymes, acetylcholine esterase (AChE), glutathione S-transferase (GST), and α-carboxyl esterase (α-CarE), in larval instars of *Culex pipiens* complex at the LC_50_ concentration. In 3rd instar larvae treated with microwave-assisted selenium nanoparticles (SeNPs-MW) and gamma-assisted selenium nanoparticles (SeNPs-G), it was found that AChE activity was significantly inhibited. On the other hand, significant increases in GST and α-CarE activities were observed. Additionally, genotoxic and ultrastructure studies of midgut epithelial cells in 3rd instar larvae revealed DNA damage and cell lysis, including destruction of the cell membrane, microvilli, and nuclei. These findings suggest that SeNPs have an adverse effect on AChE gene expression, resulting in its downregulation. This downregulation can be attributed to the formation of reactive oxygen species induced by SeNPs that can modulate the host defense mechanism leading to apoptosis and subsequent larval mortality. The present study was the first to use phyto-microwave-assisted and gamma-assisted synthesis of SeNPs which provides an eco-friendly and cost-effective solution to reduce the risk of chemical insecticides. Furthermore, an integrated pest management program (IPM) using nanocides can be successfully developed for mosquito control.

## Introduction

Mosquitoes are blood-feeding insects and are major vectors for disease transmission. In Egypt, the Rift Valley fever virus and West Nile virus are two important emerging arboviruses transmitted by *Culex pipiens* complex [1]. Furthermore, millions of people worldwide are threatened by filariasis and dengue fever because they are vectors of devastating parasites and pathogens [2]. There are still many challenges to be addressed among mosquito control programs, including insecticide resistance that develops in target vector populations.

Nanotechnology has displayed a promising and vital role in the research area that has revolutionized insect control. Thereafter, green synthesis of nanoparticles (NPs) is the most preferred and promising approach than physical and chemical preparation methods and has attracted great attention for mosquito control [3]. So, the application of these biodegradable and biosynthesized nanoparticles using plant materials as the best substitutes for chemical insecticides has emerged [4].

Selenium is considered a trace element, and it is one of the most important elements necessary for the living cells of plants and animals [5]. The use of selenium nanoparticles (SeNPs) is recommended by researchers in different scientific disciplines due to their low toxicity and high stability [6]. The eco-friendly SeNPs synthesized by naturalistic organic molecules as capping agents are kept stable and polydispersed for an extended time [7]. SeNPs have shown larvicidal activities and can be applied to decrease mosquito infections [2]. Nowadays, SeNPs are applied in mosquito bio-control [8].

Moreover, the phyto-radio synthesis of SeNPs provided an environmentally friendly, inexpensive, clean, and safe solution to control *Cx. pipiens* mosquitoes and reduce the hazards of chemical pesticides [9]. The plants are widely spread in Egypt and inexpensive as *Cupressus* *sempervirens*. The formed nanoparticles are biodegradable and leave no harmful residues in the environment. The plant extract with radiation assistance played an important role in the preparation of SeNPs, reducing selenium ions and thereby stabilizing their NPs in the prepared solution. The authors have shown that the penetration and subsequent accumulation of SeNPs through light microscopy and ultrastructure studies resulted in cell lysis and finally death.

The insect resistance can affect the efficacy of SeNPs; therefore, investigating the mode of action of these nanoparticles is essential to examine the major biomarker target enzymes, which play a vital role in the detoxification mechanism of *Cx. pipiens*. Like most insecticides, SeNPs are supposed to be detoxified by acetylcholinesterase (AChE) which hydrolyzes the neurotransmitter acetylcholine producing choline and acetic acid at the synapse [10].

Moreover, carboxylesterase (CarE) is the major enzyme that participates in the degradation of neurotransmitters and the metabolism of pheromones and hormones [11]. Glutathione S-transferase (GST) is responsible for the development of insecticide resistance and the detoxification of toxic substances [12].

Comet assay is considered an efficient tool to identify DNA damage in eukaryotic organisms at the cellular level. This technique is sensitive and cost-effective. Notably, studies on DNA damage in insects exposed to NPs are limited. DNA damage was investigated in *Aedes aegypti* larvae treated with biosynthesized silver nanoparticles (AgNPs) [13].

Several studies have shown that green synthesized SeNPs may cause significant midgut abnormalities and affect mosquito larval development. SeNPs induced histopathological malformations in the midgut of *Ae. aegypti* and *Cx. quinquefasciatus*, including midgut cell layer disorganization and peritrophic membrane damage [14]. In addition, similar histopathological damage was observed in the midgut cells of *Ae. albopictus* treated with green synthesized SeNPs [14].

The pivotal goal of the present study is to examine the cytotoxicity, neurotoxicity, and genotoxicity of phyto-microwave-assisted and gamma-assisted SeNPs as a novel approach on the larval stage of *Cx. pipiens* complex and the cellular mode of action as well as AChE gene expression.

## Materials and Methods

### Insect

*Culex pipiens* complex was laboratory-reared under controlled conditions at a temperature of 25 ± 2 °C, a relative humidity (R.H.) of 60–70%, and a photoperiod of 12 h light and 12 h dark in the insectary of the Atomic Energy Authority, Dokki, Egypt [9].

### Insecticides

*Cupressus sempervirens* has been used in the green synthesis of SeNPs-MW and SeNPs-G. These nanoparticles were characterized in our previous study [9].

### Chemicals

All reagents were purchased from Sigma Aldrich Company, Saint Louis, USA.

### Experimental Design and Tissue Preparation

Newly molted, 5 days, 2^nd^, 3^rd^, and 4^th^ larval instars were collected and treated with the LC_50_ of SeNPs-MW with sizes ranging from 11 to 55 nm and SeNPs-G with sizes ranging from 21 to 75 nm. The LC_50_ concentrations were obtained from SeNPs-MW treatment of the 2^nd^, 3^rd^, and 4^th^ instar larvae being 44.51, 28.25, and 37.60 mg/L, respectively. The LC_50_ concentrations were obtained from SeNPs-G treatment of the 2^nd^, 3^rd^, and 4^th^ instar larvae being 98.03, 31.28, and 53.58 mg/L, respectively. The LC_50_ values were based on the previous investigation of Fadl et al. [9]. Surviving larvae were homogenized in 5 mL of 0.1 M cold potassium phosphate buffer (pH 7.2). Then, 0.5 g of the tissue was centrifuged at 8000 rpm for 15 min at 4 °C; the clear supernatants were transferred to new tubes and used for the estimation of detoxifying enzyme activities. Three replicates were used for each experiment.

### Determination of Detoxifying Enzyme Activities

#### Acetylcholinesterase Activity

Acetylcholinesterase (AChE) activity was estimated using the modified Ellman procedure [15]. This method overcomes the low enzymatic specific activity because of the interfering reaction of the free SH group of proteins with 5,5′-dithiobis-2-nitrobenzoic acid (DTNB). Cholinesterase is incubated for a time interval with acetylthiocholine, and the reactions are stopped with DTNB-phosphate-ethanol reagent for yellow color development. A volume of 150 µL of 0.1 M phosphate buffer (pH 7.6) was added to 50 µL of 1 mM substrate (ASChI) and 100 µL of enzyme supernatant; this mixture was incubated for 10–15 min at 30 °C, and then, 1.8 mL of the stopping reagent (DTNB-phosphate-ethanol reagent) was added and vortexed for 10 s. The absorbance was measured by a spectrophotometer (specs) at 412 nm against blank. The mean levels of AChE activity were based on the total protein standard curve. Using a standard curve for bovine serum albumin, the total protein content was calculated using the Bradford method [16].

#### Glutathione S-Transferase (GST) Activity

The activity of GST was determined according to Habig et al. technique [17]. In this method, GST catalyzes the conjugation of the thiol group of glutathione to the substrate 1-chloro-2,4-dinitrobenzene (CDNB) giving a yellow color, GS-DNB conjugate, at 340 nm. Briefly, the reaction solution contained 50 µL of CDNB (50 mM) which was added to 150 µL of GSH (1 mM) and 2.79 mL phosphate buffer (40 mM, pH 6.8), and then, 10 µL of enzyme supernatant was added. This mixture was incubated for 2–3 min. at 20 °C. The increase in absorbance was recorded each minute for 5 min at 340 nm.

#### α-Carboxylesterase (α-CarE) Activity

The activity of α*-*CarE was assayed via the Van Aspreren et al. method [18] using α-naphthyl acetate as a substrate. The enzyme supernatant (150 µL) was incubated with 750 µL α-naphthyl acetate (0.3 mM) for 30 min at 27 °C. Then, 150 µL of the stopping solution diazo-blue lauryl sulfate (DBLS) coupling reagent was added to the mixture prepared by adding 1% FBS and 5% SDS in 2:5 v/v ratio. A red color develops immediately and is quickly changed into a stable blue color. The absorbance was recorded at 600 nm against blank. Based on protein content and α-naphthol standard curves, the means of α-CarE activities were estimated.

### Molecular Assay

#### Quantification of AChE Gene Expression

Total RNA was isolated from 3rd instar larvae treated with SeNPs-MW and SeNPs-G at LC_50_, and the larvae were preserved in 0.5 mL of RNA-later. RNA was extracted in accordance with the protocol of the QIAamp RNeasy Mini kit (Qiagen, GmbH, Catalogue no. 74104). RNA integrity was detected by formamide gel electrophoresis. Prior to cDNA synthesis, deoxyribonuclease I was used to digest total RNA (RNase-free DNase I, Fermentas, Thermo Scientific, USA). In the reaction mixture of 20 μL, RNA was reverse transcribed using Revert Aid Reverse Transcriptase Thermo Fisher kit** (**Cat. No. K1622). The expression of the target gene’s mRNAs was measured using Real-Time PCR and SYBR Green, with *Culex* ribosomal protein S7 (CPIJ006763-RA) gene as an internal reference. Stratagene MX3005P device was used for real-time quantification (Agilent Technologies, GmbH) employing the Quantitect SYBR Green PCR kit (Cat. No. 204141). Based on the gene sequences, acetylcholinesterase gene-specific primers of *Cx. pipiens* were designed according to the NCBI guidelines, and the primers were designed using primer 3 version 4.1.0, online software, based on mRNA sequences (https://primer3.ut.ee/). The primers were obtained from Eurofins Genomics, Germany, GmbH, and the expression was normalized to *Culex* ribosomal protein S7 (CPIJ006763-RA) gene (housekeeping gene) [19] (Table 1). The reactions were carried out in a 25 μL volume mixture containing 12.5 μL 2 × SYBR Green PCR Master Mix, 2 μL cDNA, 1 μL of each primer, and 8.5 μL of RNase-Free Water. PCR program was carried out with cycling conditions; each cycle (40 cycles) consisted of 5 min at 94 °C for denaturing, 30 s at the proper annealing temperature for annealing, and 30 s at 72 °C for polymerization. The dissociation stage was added after the amplification to verify the specificity of the PCR products. Stratagene MX3005P software, threshold cycle (Ct) values were measured in order to conduct quantitative analysis. Also, variations in gene expression on RNA among the different samples were assessed according to the “ΔΔCt” method, as clarified by Yuan et al. [20].

**Table 1:** Forward and reverse primer sequences for *Cx. pipiens* AChE, ace, and *Culex* ribosomal protein S7 genes

Gene	Primer sequence (5′-3′)	
*Cx* ribosomal protein S7 (housekeeping)	F	AGAACCAGCAGACCACCATC
	R	ACCCTCCCACTTCTCCATCT
*Cx* acetylcholinesterase (ace)	F	CGGACGACACTATCTGGAGC
	R	TGAGCTGCTTTCGCAAGGTA

#### Single-Cell Gel Electrophoresis (Comet Assay)

Comet assay was used to assess DNA strand breaks and was performed according to Kirilova et al. [21], with minor modifications, after treatment of 3^rd^ larval instar with SeNPs.

Two treated groups, SeNPs-MW and SeNPs-G at LC_50_, were conducted for the 3^rd^ instar *Cx. pipiens* complex larvae compared to the control group. One gram of survived larvae, control, and 5-day treated groups was washed with deionized water twice, preserved in petri dishes, and stored at − 20 °C, with needle teasing in 1 X phosphate-buffered saline (PBS), pH 7.4. After teasing for 30 s, cell homogenates were suspended in 110 µL of 1% molten low melting-point agarose (65 °C); it was placed on a microscope slide previously covered with a layer of 0.8% regular melting-point agarose. Then, agarose was gelled at 4 °C and the slide was then immersed for 24 h at 4 °C in a fresh lysis solution (164 g NaCl, 37 g of EDTA, 1 g Tris-Base merged into 890 mL of distilled water, stirred before adding 8 g of NaOH, pH 10; freshly prepared 1% TritonX-100 and 10% dimethyl sulfoxide). After lysis, slides were washed twice with distilled water and immersed for 5 min at 4 °C in a freshly prepared alkaline electrophoresis buffer (30 mL of 10 N NaOH, 0.5 mL of 200 mM EDTA into 1000 mL of distilled water, pH adjusted to 13.0 with 2 M HCl) to allow DNA unwinding and damage of alkali labile. Then, an electric field at 20 V was applied for 20 min to draw DNA negatively charged toward an anode. Afterward, slides were immersed in a neutralizing buffer for 15 min (Tris-Base, pH adjusted to 7.5 with 2 M HCl). Slides were drained, exposed to cold absolute ethanol for 5 min, stored in dry conditions, and stained with 40 µL of ethidium bromide solution. Then, the slides were examined and photographed to evaluate DNA damage using Lx 400, Labomed, USA, which is linked to a CCD camera to measure the length of DNA migration (tail length) according to this equation:

$$\mathrm{Tail}\;\mathrm{moment}\;\%\;\mathrm{DNA}\;\mathrm{in}\;\mathrm{Tail}\;\times\;\mathrm{tail}\;\mathrm{length}.$$

The % DNA content in the heads and tails was quantified by image analysis (Table 2).

**Table 2:** DNA damage analysis, assessed as tail DNA %, tail length (TL), and tail moment (TM) in the body cells of the 3^rd^ instar larvae of *Cx. pipiens* complex after 5 days of treatment with SeNPs at concentration LC_50_

Concentration (mg/L)	Head DNA (%)	Tail DNA (%)	Tail length (µm)	Tail moment
Control	92.44 ± 1.05^a^	7.55 ± 1.05^c^	2.46 ± 0.47^c^	0.50 ± 0.07^c^
SeNPs-MW	82.14 ± 2.10^b^	17.86 ± 2.10^b^	4.79 ± 0.37^b^	1.06 ± 0.11^b^
SeNPs-G	65.67 ± 2.69^c^	34.32 ± 2.69^a^	6.60 ± 0.33^a^	1.51 ± 0.04^a^

The value represents the mean ± SE of three replicates. Values in the same column with a different superscript alphabet are significantly different at *P* < 0.05

### Transmission Electron Microscopy Study (TEM)

Third-instar mosquito larvae were exposed to SeNPs. After 5 days of treatment, the larvae were collected, washed with deionized water, and dissected under a light microscope to obtain the midgut. Two larval groups were treated with SeNPs-MW and SeNPs-G at LC_50_ concentration and compared to the control group. The midgut tissues were placed in a prefixative, 5% glutaraldehyde prepared in 0.1 M sodium cacodylate buffer for 4 h at room temperature, and then washed in a buffer. The specimens were fixed by immersion in 1% osmium tetroxide prepared in 0.1 M sodium cacodylate buffer for 2 h in the dark. Fixed specimens were washed in buffer and then dehydrated through a graded ethanol series, 30%, 50%, 70%, 90%, and finally absolute ethanol. Specimens were kept in each concentration for 15 min at room temperature and in pure acetone for 30 min. Specimens were infiltrated with resin mixture; after that, the resin was polymerized at 60 °C for 48 h and then it was allowed to reach room temperature. Ultrathin sections, 50–80 nm thick, were cut with glass knives using a Leica ultracut UCT ultramicrotome, and the sections were floated on the surface of water in a ribbon shape. The sections were then flattened with chloroform vapor and manipulated to pick up onto the grids, and sections were then double stained in uranyl acetate for 15 min followed by lead citrate for 20 min [22]. Stained sections were examined by a JEOL-JEM 1010 TEM, operated at 80 kV; images were taken at different magnifications.

### Statistical Analysis

The data are shown as mean ± SE. All statistical analyses were conducted using one-way analysis of variance (ANOVA). Significance was set at *P* < 0.05, and the means were compared by Duncan’s post hoc test, using SPSS software version 21.

## Results

### Enzymes’ Activities

**Fig. 1:**
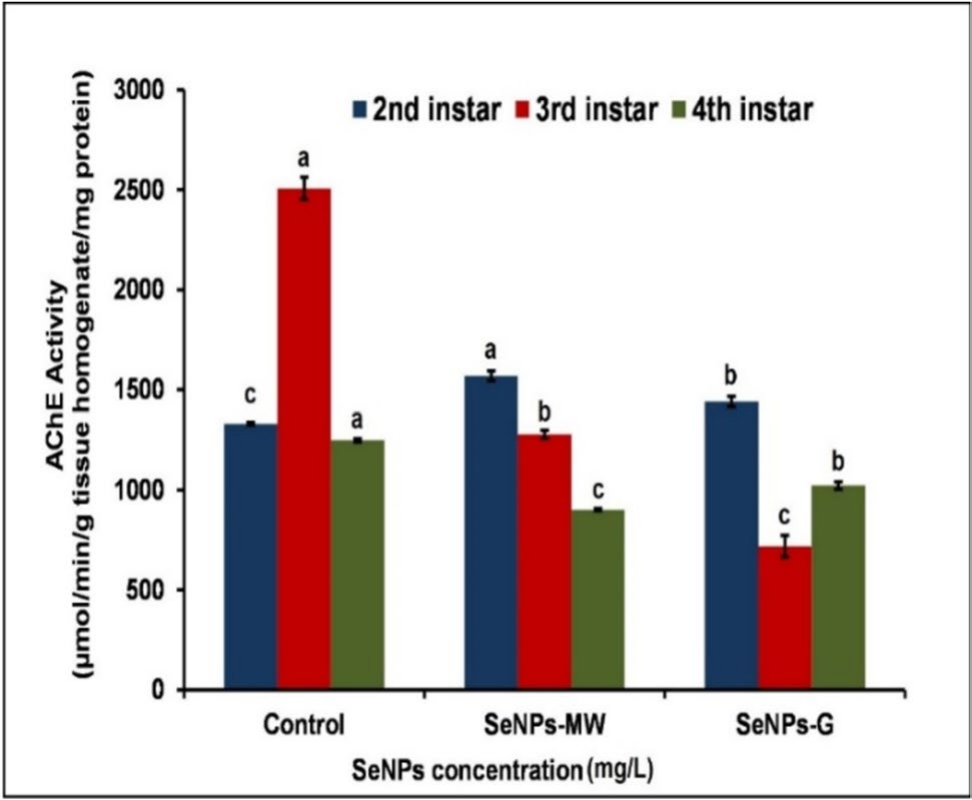
Effect of SeNPs at a concentration of LC_50_ on AChE activity in 2nd, 3rd, and 4th instar larvae of *Cx. pipiens* complex. Data are presented as mean ± SE. The means marked with different letters are significantly different in AChE activities (*P* < 0.05), whereas those marked with similar letters are not significantly different

Data presented in Fig. 1 indicated the effect of SeNPs-MW and SeNPs-G at the concentration LC_50_ on activity of AChE in 2^nd^, 3^rd^, and 4^th^ instar larvae of *Cx. pipiens* complex treated for 5 days. Significant increases in the activity of AChE by 19.75% and 11.11% were observed above the control level in 2^nd^ instar larvae treated with concentration LC_50_ of SeNPs-MW and SeNPs-G, respectively.

Treatment of the 3^rd^ instar larvae with SeNPs-MW and SeNPs-G at the concentration LC_50_ showed a significant decrease in AChE activity by 48.19% and 70.88% below the control level after 5 days, respectively. The treatment of the 4^th^ instar larvae with SeNPs-MW and SeNPs-G at the concentration LC_50_ revealed a significant decrease in AChE activity by 27.52% and 17.42% below the control level, respectively.

**Fig. 2:**
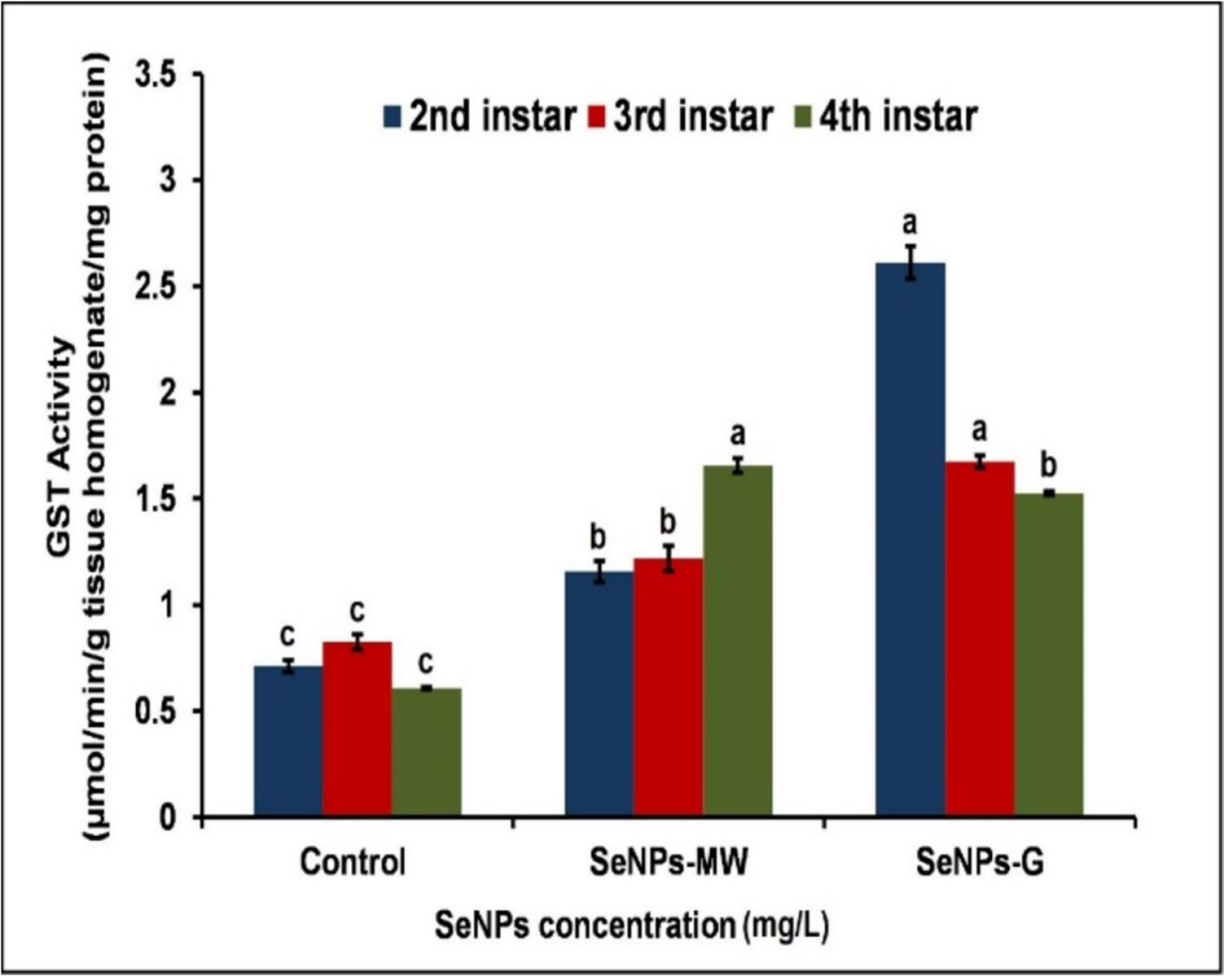
Effect of SeNPs at concentration LC_50_ on GST activity in 2^nd^, 3^rd^, and 4^th^ instar larvae of *Cx. pipiens* complex. Data are presented as mean ± SE. The means marked with different letters are significantly different in GST activities (*P* < 0.05), whereas those marked with similar letters are not significantly different

Figure 2 demonstrates a significant increase in GST activity in the 2^nd^ instar larvae treated with SeNPs-MW for 5 days at the LC_50_ concentration, showing a remarkable enhancement of 65.08% compared to the control level. Similarly, treatment with the LC_50_ concentration of SeNPs-G resulted in a substantial increase in GST activity, reaching 276.11% higher than the control level. In the 3^rd^ instar larvae, both SeNPs-MW and SeNPs-G at the LC_50_ concentration significantly elevated GST activity after 5 days, with percentage differences of 49.94% and 106.11% above the control level, respectively. Furthermore, in the 4th instar larvae treated with the LC_50_ concentration for 5 days, SeNPs-MW and SeNPs-G exhibited significant increases in GST activity by 173.66% and 152.68%, respectively.

**Fig. 3:**
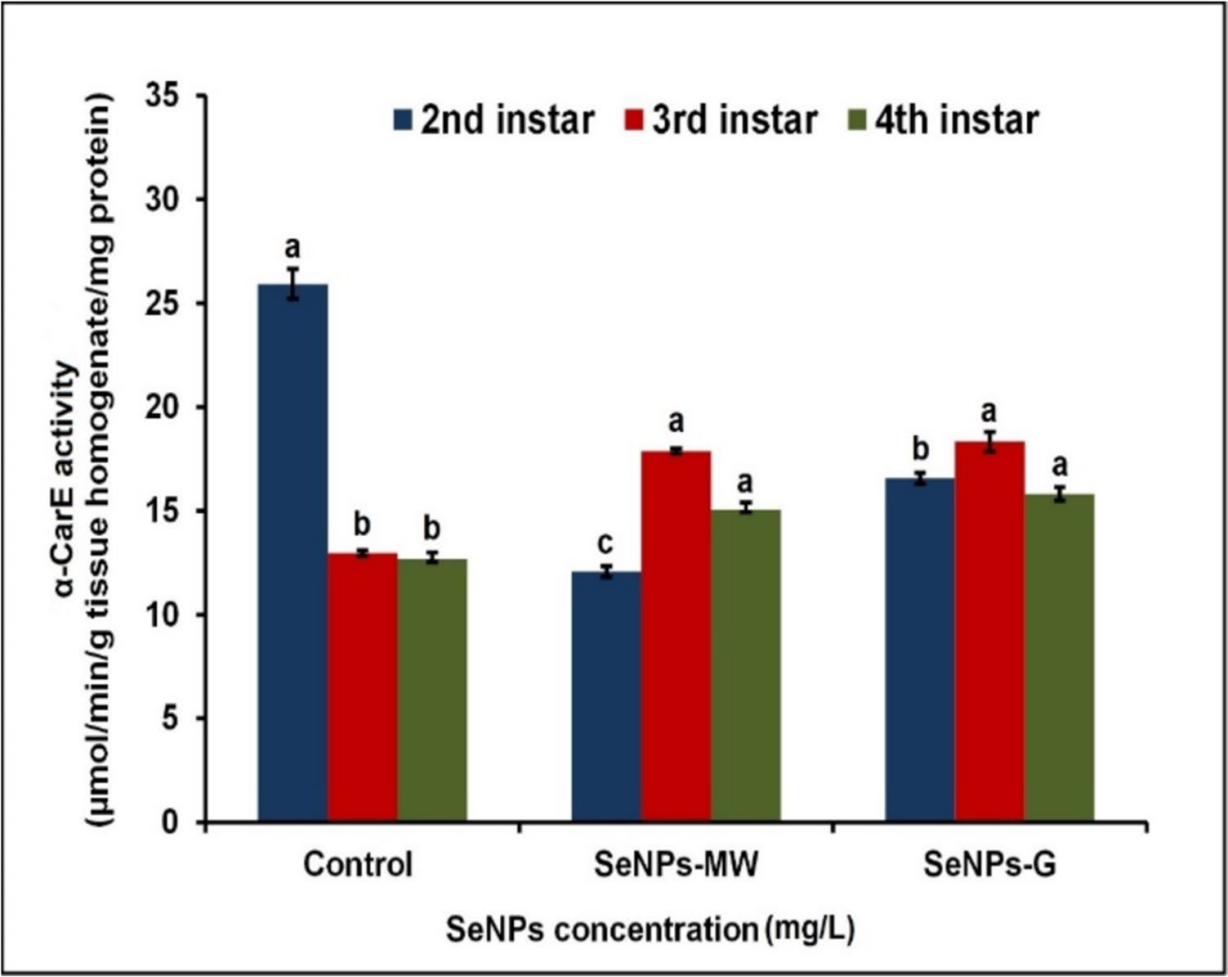
Effect of SeNPs at concentration LC_50_ on α-CarE activity in 2^nd^, 3^rd^, and 4^th^ instar larvae of *Cx. pipiens* complex. Data are presented as mean ± SE. The means marked with different letters are significantly different in α-CarE activities (*P* < 0.05), whereas those marked with similar letters are not significantly different

SeNPs-MW-treated 2^nd^ instar larvae at concentration LC_50_ induced a significant decrease in α-CarE activity by 47.04% below the control level after 5 days of treatment. Moreover, α-CarE activity decreased significantly by 28.84% after SeNPs-G treatment at the LC_50_ concentration (Fig. 3). α-CarE activity revealed significant increases in the 3^rd^ instar larvae treated with SeNPs-MW and SeNPs-G at the LC_50_ concentration by 37.58% and 41.01% above the control level, respectively, after 5 days of treatment. Moreover, the effect of SeNPs-MW and SeNPs-G showed significant increases in α-CarE activity in the 4th instar larvae after 5 days of treatment by 17.46% and 23.54% compared to the control level, respectively.

### AChE Gene Expression

**Fig. 4:**
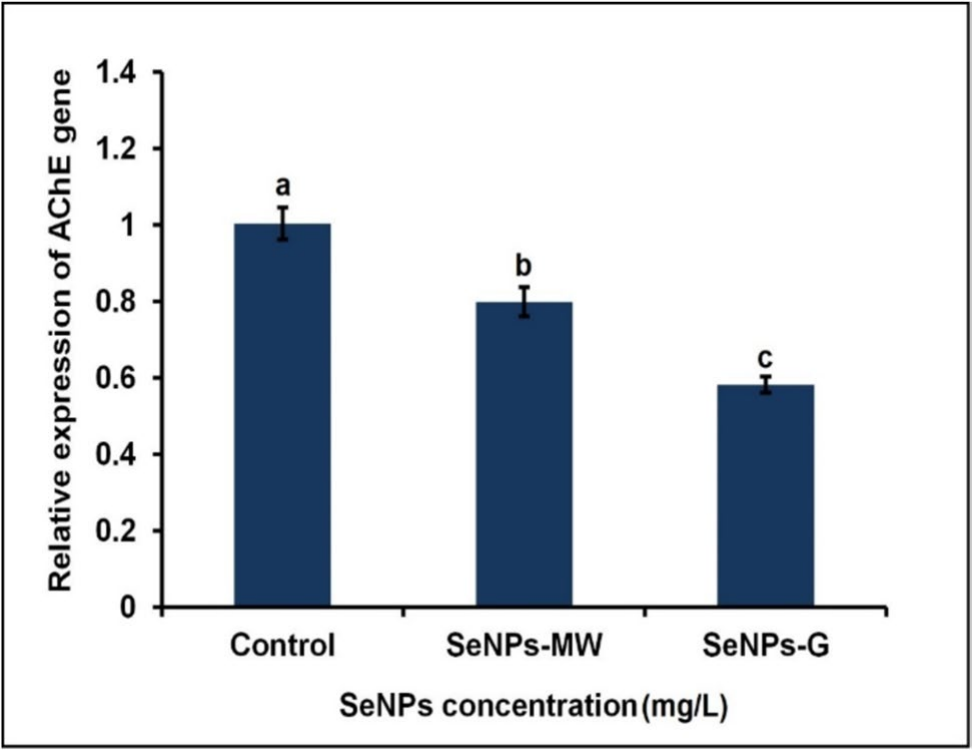
Expression of AChE gene in the 3^rd^ instar larvae of *Cx. pipiens* complex after 5 days of treatment with SeNPs at concentration LC_50_. Data are presented as mean ± SE. The means marked with different letters are significantly different (*P* < 0.05)

A significant decrease in the gene expression of AChE by 20.42% was observed in the 3^rd^ instar larvae of *Cx. pipiens* complex treated with SeNPs-MW at the LC_50_ concentration for 5 days compared to control (Fig. 4). Similarly, treatment of the larvae with SeNPs-G at the LC_50_ concentration revealed a significant decrease of AChE gene expression level by 42.03% after 5 days of treatment compared to control. The results of the RT-qPCR confirmed the downregulation of the enzyme activity and further mortality concur to which may underlie the inhibition of AChE activity in the 3^rd^ instar larvae.

### Single-Cell Gel Electrophoresis (Comet Assay)

**Fig. 5:**
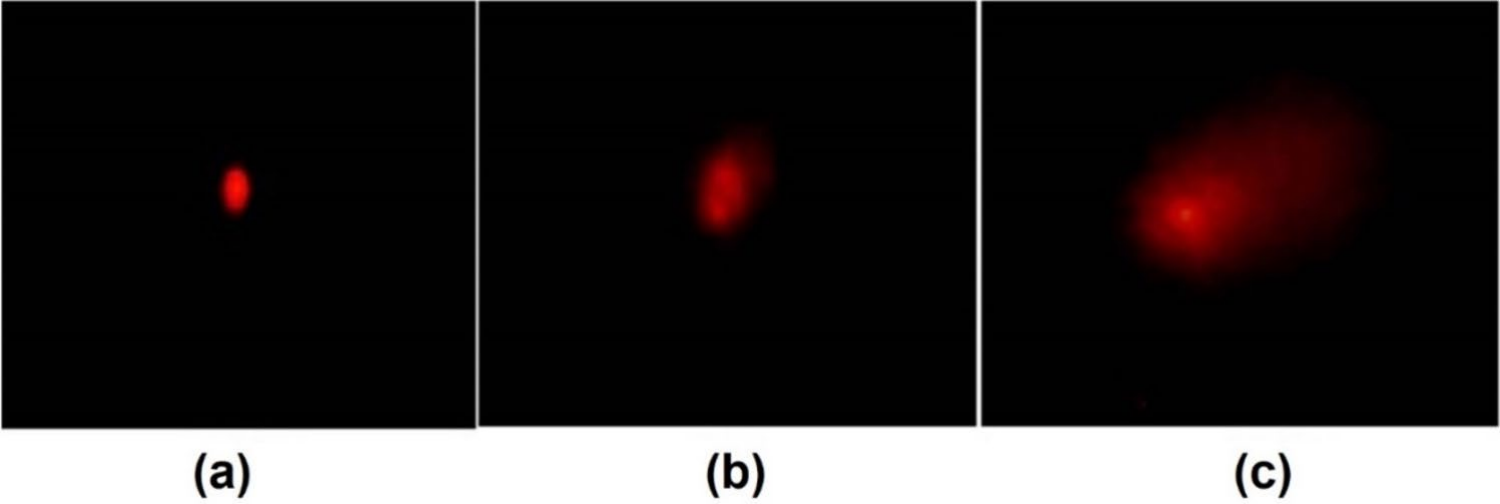
Different cell damage by using comet assay. **a** Control. **b** SeNPs-MW. **c** SeNPs-G

**Fig. 6:**
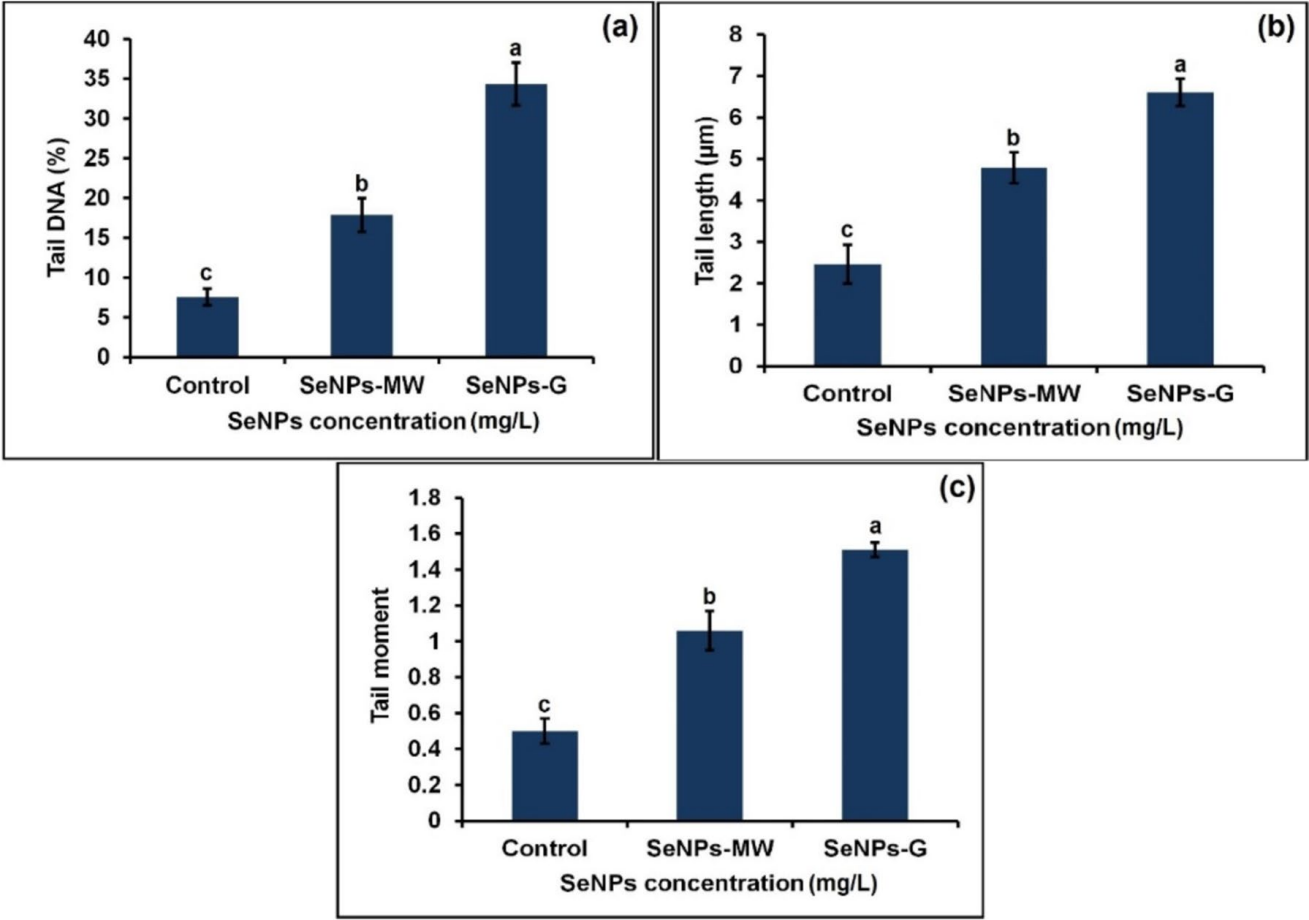
DNA damage analysis, assessed as **a** tail DNA %, **b** tail length (TL), and **c** tail moment (TM) in the body cells of the 3^rd^ instar larvae of *Cx. pipiens* complex. Data are presented as mean ± SE. The means marked with different letters are significantly different (*P* < 0.05)

The assessment of DNA fragmentation in the cells of the 3^rd^ instar larvae of *Cx. pipiens* complex treated with SeNPs at concentration LC_50_ is represented in Fig. 6 where the levels of DNA fragmentation are shown as tail DNA %, tail length (TL), and tail moment. The tail moment values were used as an arbitrary expression for the quantitative measurement of DNA strand-breaks. Both SeNPs-MW and SeNPs-G induced DNA fragmentation at concentration LC_50_ as indicated by the presence of DNA tail. The nuclei in the body cells of the control group appeared almost rounded, while the nuclei showed a tail-like extension after treatment of the 3^rd^ instar larvae with SeNPs-MW and SeNPs-G (Fig. 5). DNA fragmentation of the body cells was analyzed by comet assay (Fig. 6) which showed the comet length significantly higher than the control in case of SeNPs-MW (4.79 µm) and SeNPs-G (6.60 µm). Moreover, it was found that the two treatments, SeNPs-MW and SeNPs-G, caused significant increase in the value of tail moment (TM) (1.06 and 1.51, respectively). DNA damage in the SeNPs-MW and SeNPs-G-treated 3^rd^ instar larvae at LC_50_ showed a percentage increase of tail DNA by 17.86% and 34.32%, respectively, compared to control.

### Transmission Electron Microscopy Study (TEM)

**Fig. 7:**
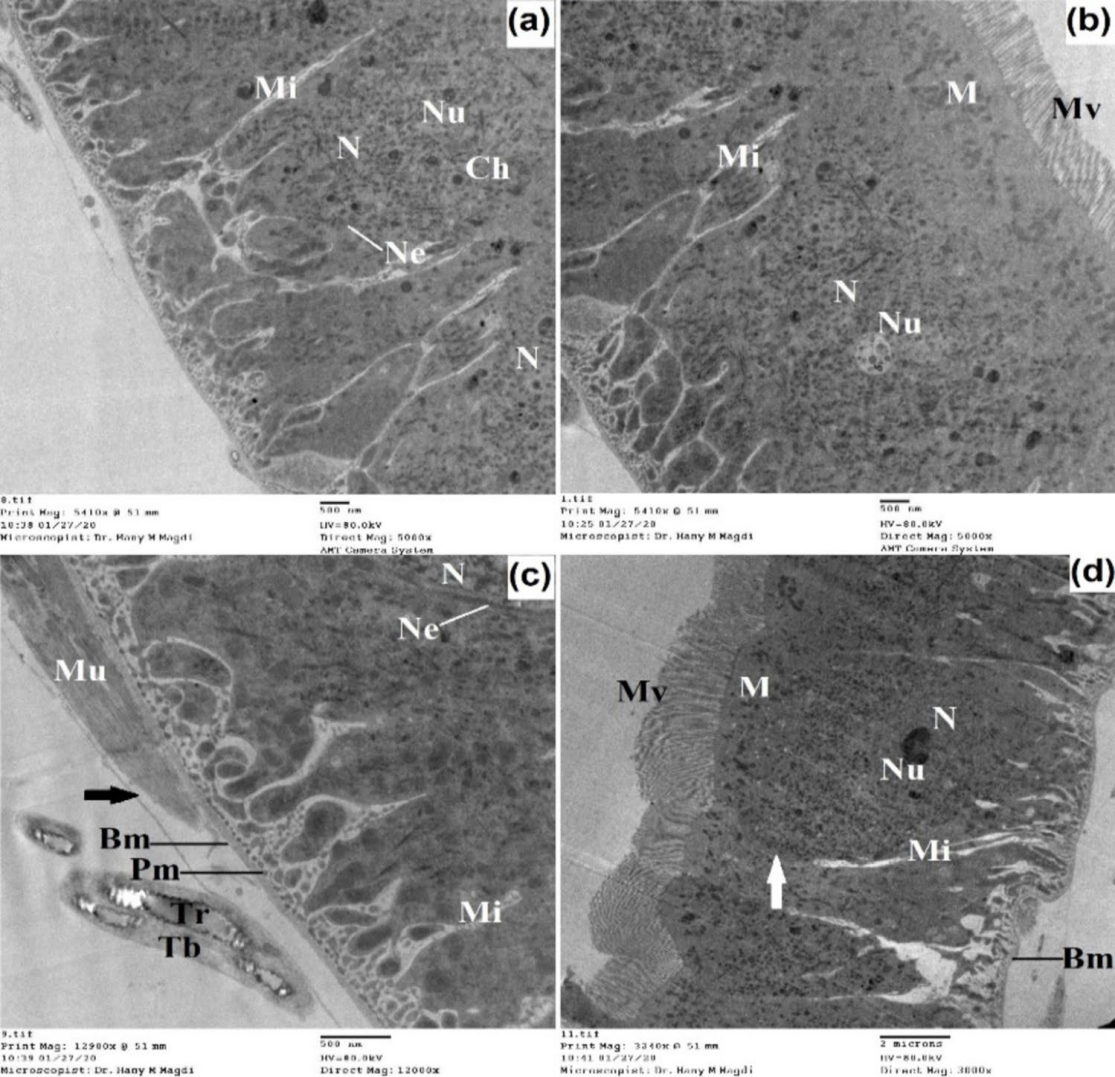
Transmission electron micrograph of the midgut of untreated 3^rd^ instar larvae of *Cx. pipiens* complex showing well-developed epithelial cells resting on the basement membrane and surrounded by circular muscle bundles. The columnar cells appeared with large nuclei that exhibited condensed chromatin and developed nucleolus. The outer membrane appeared with regular projections called microvilli. Septate junction (white arrow), organ investment layer (black arrow); basement membrane (Bm), chromatin material (Ch), membrane invagination (Mi), microvilli (Mv), mitochondria (M), muscles (Mu), nuclear envelope (Ne), nucleolus (Nu), nucleus (N), plasma membrane (Pm), tracheoblast (Tb), tracheole (Tr). **a**, **b** X = 5000; **c** X = 12,000; **d** X = 3000

**Fig. 8:**
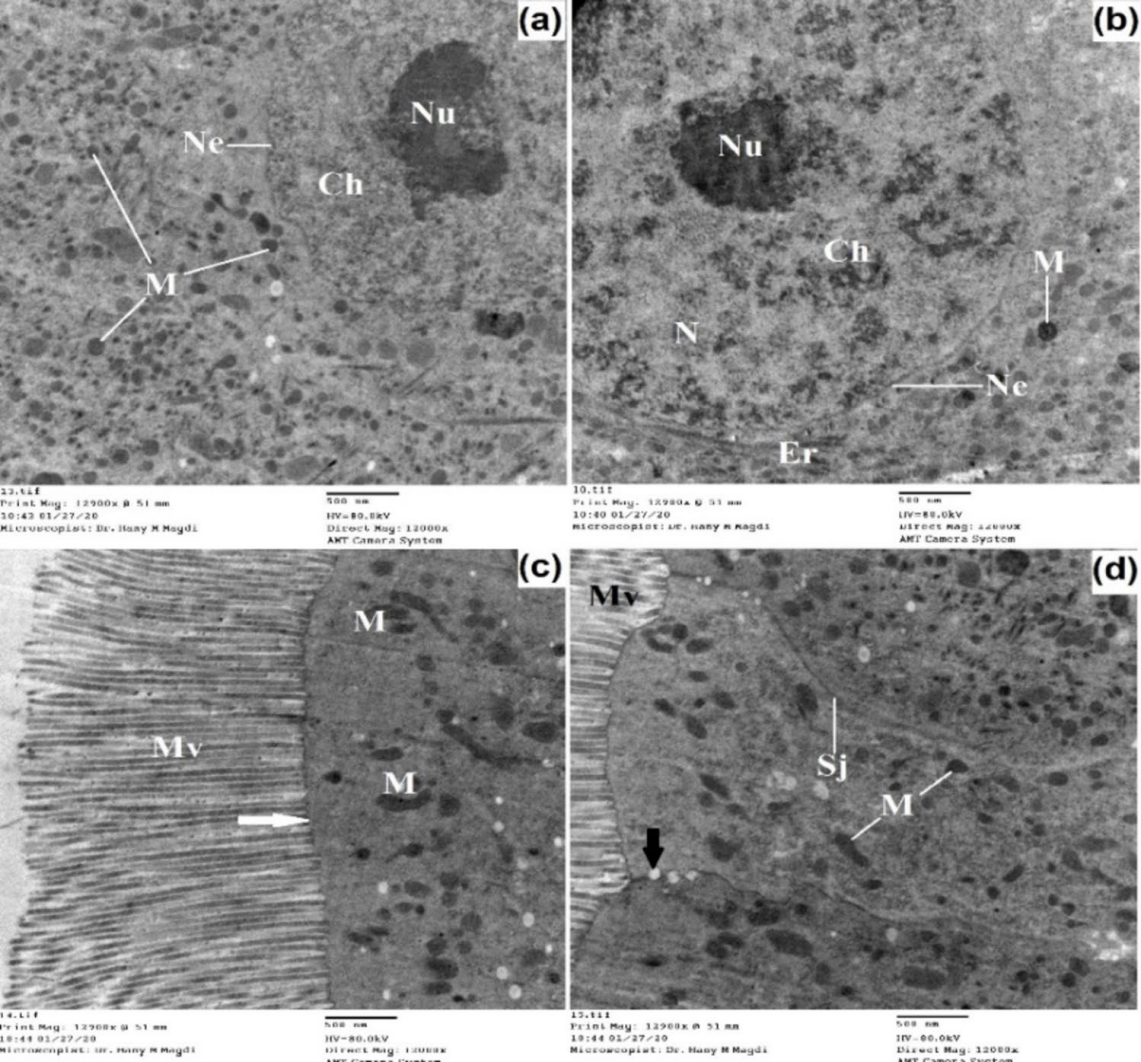
Transmission electron micrograph of the midgut of untreated 3^rd^ instar larvae of *Cx. pipiens* complex showing the nucleus, the chromatin materials, long microvilli, septate junctions, and gap junctions between epithelial cells. Large numbers of mitochondria toward the outer plasma membrane appeared obvious. Gap junctions (black arrow), outer plasma membrane (white arrow); chromatin material (Ch), endoplasmic reticulum (Er), microvilli (Mv), mitochondria (M), nuclear envelope (Ne), nucleolus (Nu), nucleus (N), septate junction (Sj). X = 12,000

The midgut of untreated 3^rd^ instar larvae has a single layer of epithelial cells surrounded by circular muscle bundles and also tracheoles are observed (Figs. 7 and 8). The midgut cells are columnar with a large spherical nucleus which is basely located. The nucleus exhibited condensed chromatin and a well-developed nucleolus; the endoplasmic reticulum was also observed.

The inner plasma membrane at the haemocoel side of the midgut epithelium has its area increased by indentation which represents the infoldings of the plasma membrane branching and penetrating a distance in the midgut epithelium.

Between the inner plasma membrane and the hemolymph, there are two membranes: one of these is the basement membrane which is closely connected with part of the inner plasma membrane. The second one is called the organ investment layer which resembles a simple basement membrane and appeared as a supporting envelope for holding muscles and trachea to the gut (Figs. 7 and 8). The outer plasma membrane appears uniform, and the surface area of this membrane is increased by well-developed regular brush borders called microvilli. The microvilli are tightly packed having internal fibers which extend as channels into the body of the cells and increase the surface area 20–50 times. Moreover, mitochondria were present in the midgut cells and were widely distributed toward the apical cytoplasm of the epithelial cells. Between the adjacent membranes of the two epithelial cells, a septate junction is observed with gap junctions at the apical part of the cell.

**Fig. 9:**
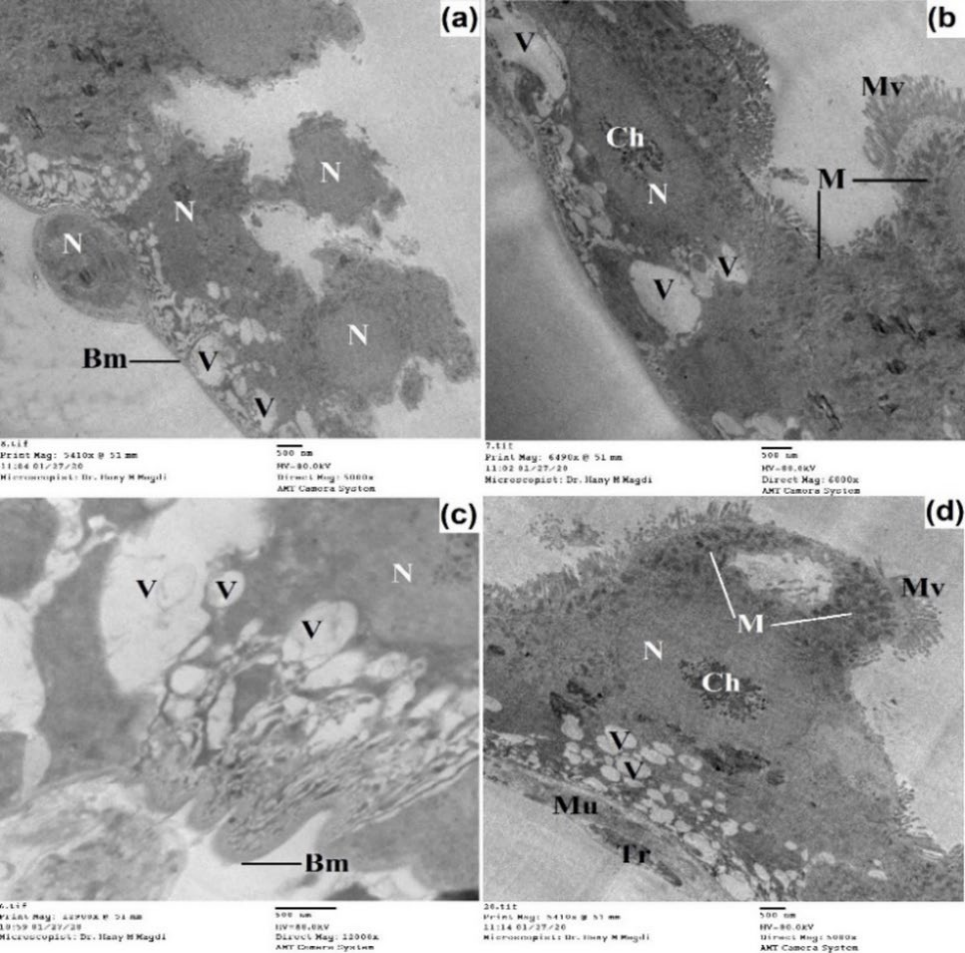
Transmission electron micrograph of the midgut of treated 3^rd^ instar larvae of *Cx. pipiens* complex with SeNPs-MW showing damaged epithelial cell layer with numerous vacuoles and reduced microvilli. Basement membrane (Bm), chromatin material (Ch), muscles (Mu), microvilli (Mv), mitochondria (M), nucleus (N), plasma membrane (Pm), tracheole (Tr), vacuole (V). **a** X = 5000; **b** X = 6000; **c** X = 12,000; **d** X = 5000

**Fig. 10:**
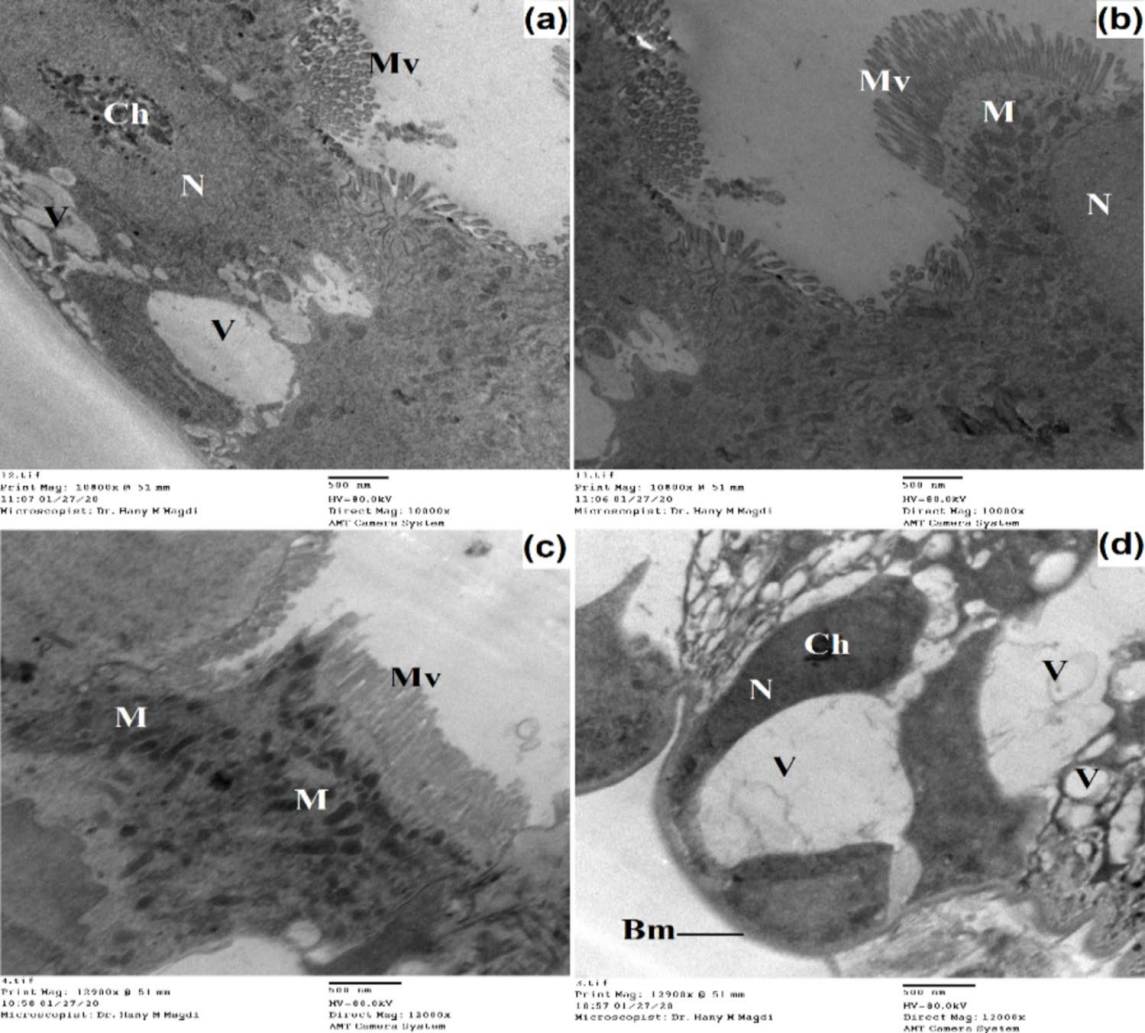
Transmission electron micrograph of the midgut of treated 3^rd^ instar larvae of *Cx. pipiens* complex with SeNPs-MW showing damaged basement membrane with several folding, elongated nucleus, several vacuoles, and shortened microvilli. Basement membrane (Bm), chromatin material (Ch), microvilli (Mv), mitochondria (M), nucleus (N), vacuole (V). **a**, **b** X = 10,000; **c**, **d** X = 12,000

Five days after the treatment with SeNPs-MW, several cytological disturbances appeared like severe damage of mitochondria, lysis of the epithelial cells, and appearance of large amounts of vacuoles in the midgut epithelium (Figs. 9 and 10). The basement membrane appeared invaginated and detached from the epithelial layer. The nucleus showed abnormalities like condensed chromatin and, being elongated, lost its spherical appearance; also, the nucleus protruded in some positions into the lumen and into the haemocoel side in other positions. The microvilli became reduced and ruptured and lost their uniformity.

**Fig. 11:**
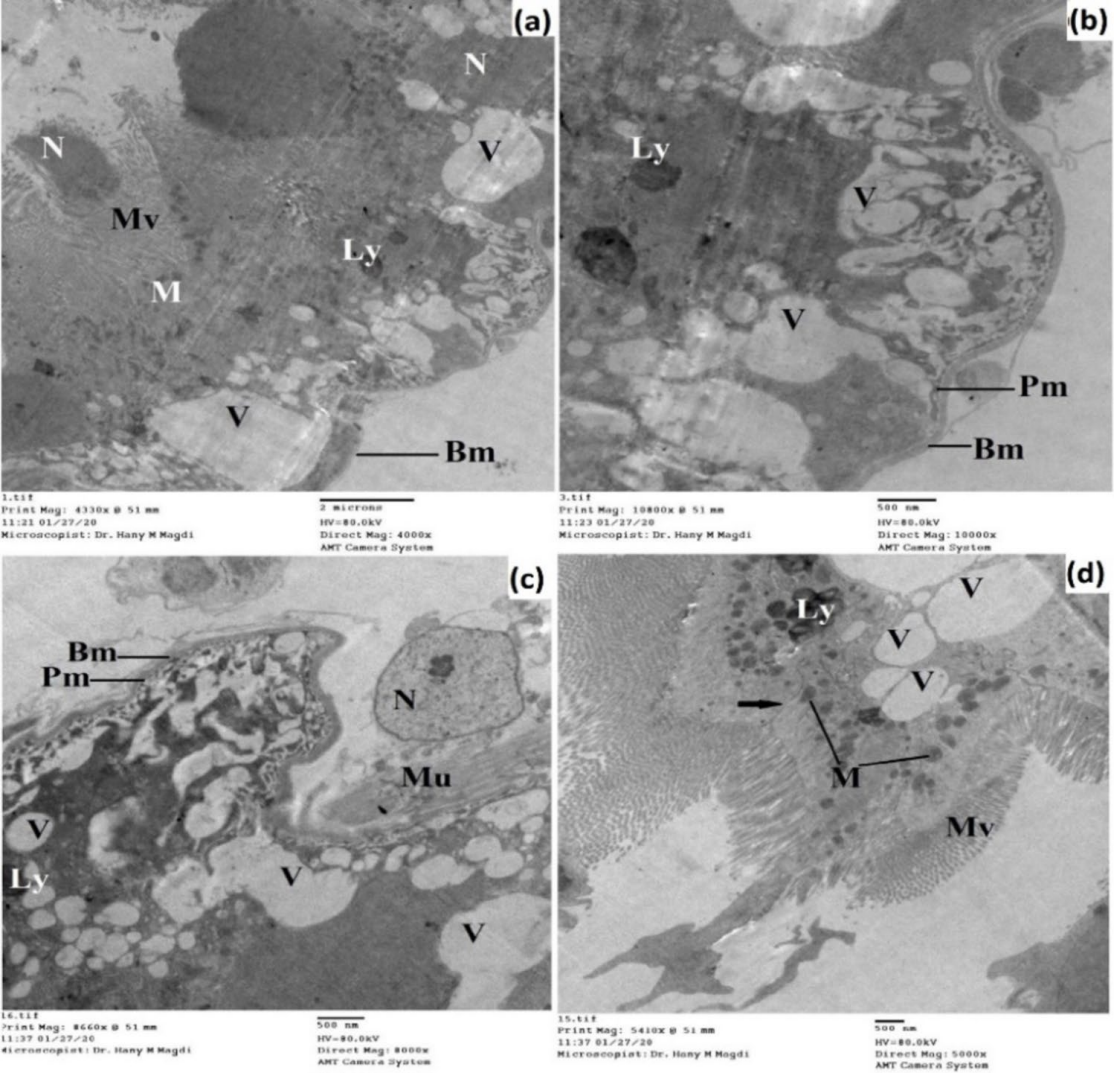
Transmission electron micrograph of the midgut of SeNPs-G-treated 3^rd^ instar larvae of *Cx. pipiens* complex showing destructed epithelial layer with migrating nucleus toward the lumen appearance of lysosomes and vacuoles. Septate junction (black arrow); basement membrane (Bm), lysosomes (Ly), microvilli (Mv), mitochondria (M), muscles (Mu), nucleus (N), plasma membrane (Pm), vacuole (V). **a** X = 4000; **b** X = 10,000; **c** X = 8000; **d** X = 5000

**Fig. 12:**
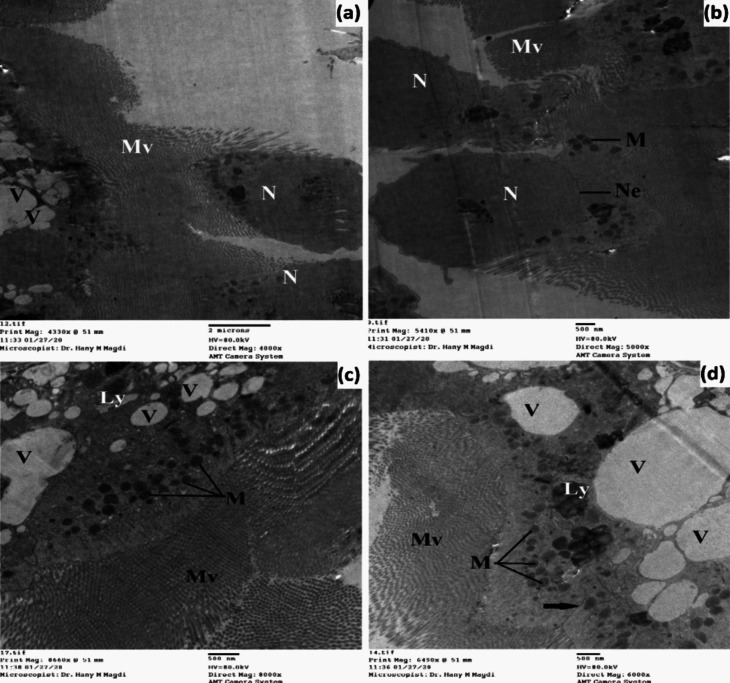
Transmission electron micrograph of the midgut of SeNPs-G-treated 3^rd^ instar larvae of *Cx. pipiens* complex showing complete damage for the epithelial cells and appearance of several vacuoles and lysosomes and shortened microvilli. Septate junction (black arrow); lysosomes (Ly), microvilli (Mv), mitochondria (M), nucleus (N), nuclear envelope (Ne), vacuole (V). **a** X = 4000; **b** X = 5000; **c** X = 8000; **d** X = 6000

Treatment of the 3^rd^ instar larvae with SeNPs-G induced several severe pathological damages in the epithelial cell layer which detached from the basement membrane (Figs. 11 and 12). Several vacuoles were distributed in the midgut cells, there was severe damage in the mitochondria, and the appearance of lysosomes were noticed. Complete rupture of the nuclear membrane was clear, and the nucleus protruded toward the midgut lumen and haemocoel. The microvilli of the epithelial cells were destroyed.

## Discussion

The enzymatic activity can be disrupted by NPs, thus increasing the production of reactive oxygen species, which decrease or inactivate the detoxifying enzyme activity [9].

The 2^nd^ instar treated samples with SeNPs-MW and SeNPs-G showed a significant increase where the 3^rd^ and 4^th^ instar larvae showed significant decrease in the activity of AChE compared to control.

The activity of the enzymes has been used as an important indicator for monitoring insect development and resistance to pesticide. This may be due to the development of defense mechanisms and resistance to SeNPs [23].

The current results match those reported by Fouad et al. [23] who demonstrated a significant decrease in AChE activity as a result of treatment with AgNPs in the 4^th^ instar of *Cx. pipiens* and *Ae. albopictus*, respectively. Similarly, Solairaj and Rameshthangam [24] showed a decrease in AChE activity in all larval instars of *Ae. aegypti* upon treatment with α-chitin NPs, AgNPs, and α-chitin/silver nanocomposite. In agreement with [25], AChE activity decreased in the 4^th^ larval instar of *Ae. aegypti* exposed to AgNPs.

AChE is responsible for termination of the nerve impulses transmitted by ACh and can bind NPs which affect its activity [26, 27]. Therefore, AChE enzyme breaks down acetylcholine which carries the transmission of nerve impulses through the synaptic gap and acts as a primary target for synthetic insecticides. Inhibition of enzyme activity is a proven method for disrupting a great number of important physiological and biochemical processes. Therefore, this enzyme is useful for determining the possible neurotoxicity of SeNPs. The authors concluded that the variation in AChE activities may be due to different susceptibilities of 2^nd^, 3^rd^, and 4^th^ instar larvae toward SeNPs.

GST enzyme is a multifunctional enzyme; it plays an important role in protecting insects from oxidative damage, as well as in antioxidant process, the detoxification of toxic substances by catalyzing the conjugation of reduced glutathione, thus rendering them less toxic [28], and helps to neutralize the toxicity of different xenobiotics. Through chemical metabolism or sequestration, GST protect against oxidative stress caused by chemicals and enable adaptability to a wide variety of xenobiotics. The structure of the various types of GSTs, namely the makeup and spatial arrangement of the amino acid residues found in the catalytic active sites, is crucial to understanding their varied functions [29].

The present study illustrated increased levels of GST activities in the 2^nd^, 3^rd^, and 4^th^ instar larvae treated with SeNPs-MW and SeNPs-G. Elevated GST activity has been related to the detoxification process in larvae and indicates a defense response and an attempt to adapt by the vector against SeNPs as it reduces the possibility of compounds binding with other macromolecules such as DNA [30]. This increment in GST activity was in accordance with Hu and Gao [26] where the enzyme level increased significantly at the higher concentrations of AgNPs in *Ae. aegypti*. Besides the detoxification role of GST, it contributes to cell protection from oxidative damage [31]. The increase in the activity of antioxidative enzymes may serve to overcome the toxicity effect of extracellular reactive oxygen species (ROS) on the larvae [32, 33].

The present study showed a significant decrease in the Car-Est enzyme activities in the 2nd larval instar while the 3^rd^ and 4^th^ instar larvae showed an increase in the enzyme activities after being treated with SeNPs-MW and SeNPs-G.

Esterases are the major enzymes responsible for resistance mechanisms to insecticides in mosquitoes by cleaving the carboxyl ester bond and phosphodiester bond [34]. The highest CarE activity observed in the 3^rd^ and 4^th^ instar larvae after treatment with both SeNPs-MW and SeNPs-G at LC_50_ concentration can be explained by increased resistance of mosquitoes to insecticides [34]. In contrast, the 2^nd^ instar larvae showed a significant decrease in α-CarE activity. This finding agrees with Adamo [32] and Gretscher et al. [33] upon treatment of the 4^th^ instar *Cx. pipiens* and *Ae. albopictus* with AgNPs. The decreased activity of esterase can degrade juvenile hormones and their analogues, thus playing a major role in reproduction [35] and causes changes in the metabolism of fats and lipids [36], and this may explain the delayed growth observed during the current study. The authors concluded also that the decrease in esterase activities in the 2^nd^ instar larvae or the increase in the 3^rd^ and 4^th^ instar larvae may be due to different susceptibilities in the different instar larvae to SeNPs. Similar results were reported that α-/β-esterases and GST have been extensively explored in different mosquito species treated with alumina NPs [28]. It has been reported that GST and CarE may have evolved resistance through direct detoxification and induce the elimination of endogenous and exogenous compounds through different metabolic pathways and/or convert them into useless substances that can be easily eliminated by the insect body [37].

In the current study, the results of the RT-qPCR confirmed the downregulation of the enzyme activity and further mortality concur to which may underlie the inhibition of AChE activity in the 3^rd^ instar larvae.

The obtained results showed a significant decrease of AChE gene expression level in the 3^rd^ instar larvae of *Cx. pipiens* of treatment with SeNPs-MW and SeNPs-G.

In mosquitoes, AChE gene is one of the resistance genes as it is the main target of many insecticides like organophosphorus (OP) and carbamates [38]. Mosquitoes carry two genes of AChE, ace-1 and ace-2 [38]. Two acetylcholinesterase, AChE1 and AChE2, are expressed in mosquitoes but there is an exception in the case of *Cx. pipiens*, as only one AChE is present with catalytic properties similar to those of AChE1 [39]. The downregulation of AChE expression has been demonstrated to rescue cells from apoptotic death and inhibition of AChE causes a decline in catalytic function, disrupting apoptotic cell death in diverse cell types [40]. Besides the major role of AChE in hydrolyzing the neurotransmitter acetylcholine at the synapses, AChE performs non-synaptic functions involved in growth regulation, development, and reproduction [41]. Moreover, Bricker et al. reported AChE expression in non-neuronal cells [42]. Knorr et al. discussed the downregulation of AChE gene expression by suggesting that the apoptotic process in locust neurons is facilitated by the catalytic activity of AChE. This is evident from the fact that cell death induced by hypoxia was prevented by AChE inhibitors. Both inhibitors have been proven to effectively inhibit insect AChE activity [43]. In this study, the authors proposed that SeNPs performed the same function in inhibiting AChE activity. In this study, the authors proposed that SeNPs performed the same function in inhibiting AChE activity.

Almost no studies have been reported on the impact of nanoparticles on the expression of AChE gene in *Cx. pipiens*. In the present study, a significant downregulation in the expression of the AChE gene by 20.4% and 42% was recorded upon treatment of the 3rd instar larvae of *Cx. pipiens* with SeNPs-MW and SeNPs-G, respectively. This result from the RT-qPCR confirmed the downregulation and further mortality concur to the biochemical results of AChE activity which inhibited in the 3rd instar larvae. Malik et al. demonstrated the downregulation of AChE gene in white flies exposed to transgenic tobacco plants as a safe way in insect management [44]. Few studies demonstrated several molecular changes when larvae were exposed to NPs [45]. The authors observed up and downregulation in the expression of glutathione S-transferase (GST) and ecdysone receptor gene, respectively, when the aquatic midge *Chironomus riparius* was exposed to different concentration of AgNPs. The ribosomal protein gene (CrL15) was also downregulated when *Chironomus riparius* was treated with AgNPs [46]. Nyakundi Erick et al. reported a significant decrease in the expression of GPX gene and increase in the expression of CAT and SOD genes in *Anopheles stephensi* under the effect of AgNPs [47].

Comet assay has been used to identify DNA damage and has been applied in genotoxicity [13]. SeNPs-MW and SeNPs-G-treated 3rd instar larvae at LC_50_ showed percentage tail DNA 17.86% and 34.32%, respectively. These results may be explained by the ability of SeNPs to induce ROS generation which resulted in oxidative stress leading to severe DNA damage and finally cell death [48]. Similarly, Devi et al. showed percentage tail DNA damage of 3.18% and 31.70% after treatment of *Ae. aegypti* larvae with AgNPs at LC_50_ and LC_90_, respectively [13].

Genotoxicity data for NPs in insects are rare; Mao et al. reported that AgNPs caused accumulation of ROS in the fly tissues of *Drosophila melanogaster* which resulted eventually in apoptosis [48].

Genotoxicity of SeNPs may be mediated by the direct interaction of Se nanoparticles with DNA or other cellular constituents related to DNA integrity. SeNPs accumulating in the larval cells interact directly with the DNA inside the nucleus. The nanopesticides, SeNPs, are either transported through the pores in nuclear membrane or they are trapped inside the nucleus during the breakdown of the nuclear membrane in mitosis consequently prompting several DNA damages [49].

Many investigations have examined cellular changes in *Cx. pipiens* larval midgut caused by nanoparticles. The midgut of *Cx. pipiens* larvae is composed of a single epithelial layer with prominent large nuclei. The outer plasma membrane is provided with regular projections known as microvilli and abundant mitochondria distributed toward the apical cytoplasm [50]. Several abnormal changes were induced in the larval midgut by green synthesized SeNPs. In this study, the obtained results indicated the effects of SeNPs-MW and SeNPs-G on the midgut epithelial cells of the 3^rd^ instar larvae *Cx. pipiens* after 5 days of treatment. These intensive deteriorations in the epithelial cells were induced by the penetration of the SeNPs and their reaction with membrane protein and interaction with cellular components leading to their damage.

Similar effects were also observed in *Ae. aegypti* larvae treated with AgNPs causing degeneration of nuclei and partial lysis of midgut wall [51]. Histological sections of *Ae. aegypti* larvae treated with SeNPs revealed damages in larval midgut, peritrophic membrane, and cuticle layer compared to untreated larvae [52].

Cittrarasu et al. studied the effect of SeNPs on the midgut tissues in both *Ae. aegypti* and *Cx. quinquefasciatus* larvae, and the images depicted several damages such as cell lysis, breakage of peritrophic membrane, and disorganized and broken epithelial cells [14]. Additionally, a structural alteration in the midgut epithelial cells was detected by Cittrarasu et al. [14] in *Ae. albopictus* larvae treated with SeNPs. Sundararajan et al. reported the midgut epithelial cell damages of the 3^rd^ and 4^th^ instar larvae of *Ae. aegypti* treated with green synthesized AuNPs and their accumulation in the midgut region [53].

**Fig. 13:**
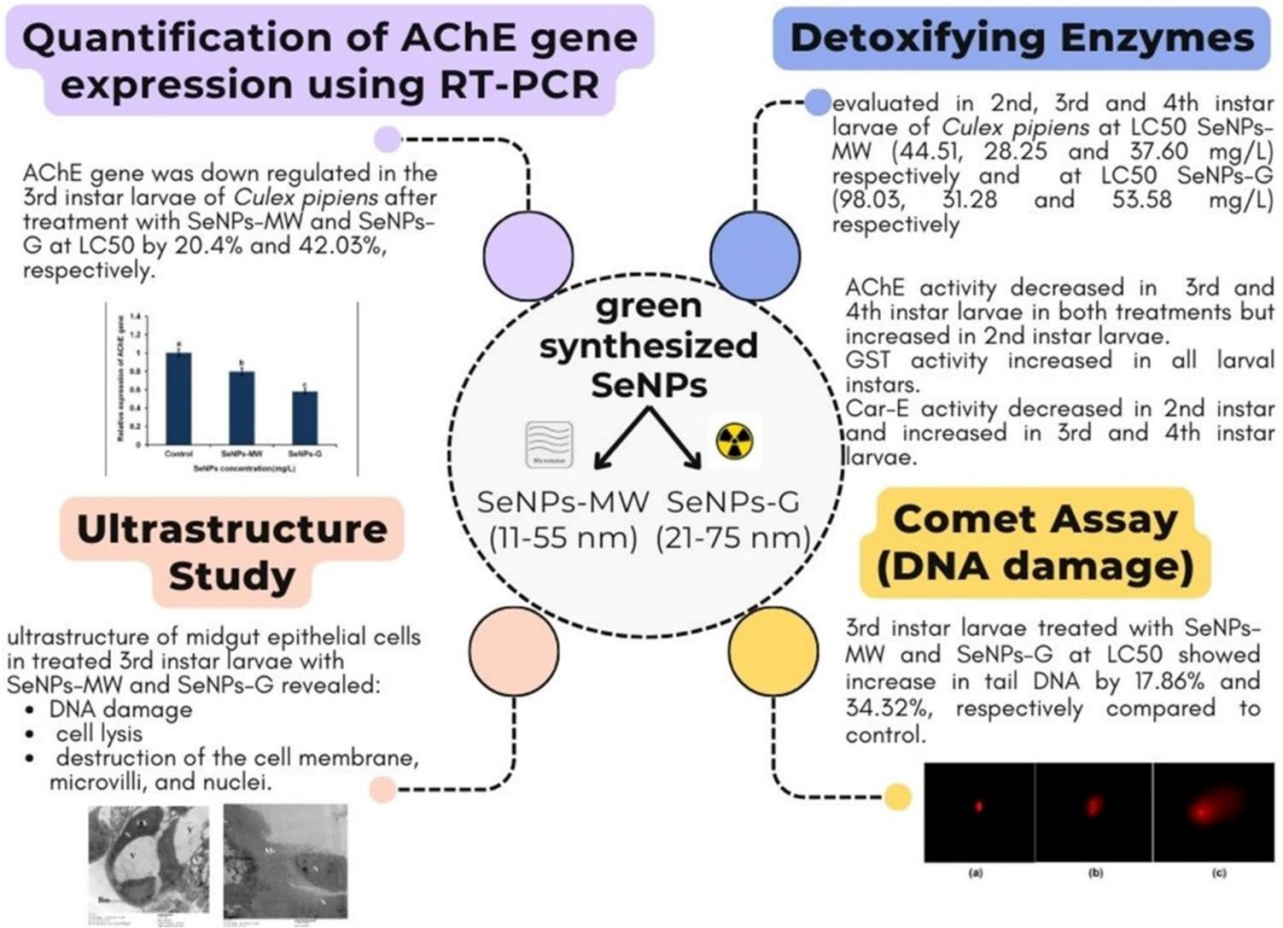
Schematic representation of all results

It can be concluded from the present results that the phyto-radio synthesis of SeNPs provides an eco-friendly and cost-effective solution to reduce the risk of chemical insecticides (Fig. 13). The susceptibility of *Cx. pipiens* complex especially the 3^rd^ instar larvae to SeNPs-MW and SeNPs-G treatments was obvious in the alterations of AChE, GST, and α-CarE activities, the effects being more prominent after SeNPs-G exposure. It should be emphasized that both SeNPs-MW and SeNPs-G treatments at the concentration LC_50_ induced a downregulation in AChE gene expression showing its neurotoxicity in larvae of *Cx. pipiens* complex. The current study provides important data on genotoxicity of SeNPs, confirming DNA damage in the treated *Cx. pipiens* complex larvae. Nanopesticides are the future alternative to chemical or biopesticides. The use of nanopesticides represents a novel strategy which is urgently needed to reduce vector-borne diseases through an appropriate control method other than the adverse methods affecting human health. The reported results may aid the development of integrated pest management programs, IPM, for mosquito control in Egypt. Moreover, the synthesized NPs are more suitable for medical applications due to the chemical-free nature of the synthesized NPs; meanwhile, the size, shape, and morphology of NPs can be controlled and mediated by plant extract. The most advantageous thing about green synthesis is no need for additional agents to synthesize and stabilize NPs like reducing agents, capping agents, and stabilizing agents.

A limitation of the present study is the application of SeNPs on a small scale that does not provide information on the success of these particles in semi-field studies and their resistance-induced or mutant effects. The lack of information on the adverse effects of these particles on non-target organisms is another limitation.

## Data Availability

No datasets were generated or analysed during the current study.
